# Cardioprotective effects of *Bassia indica* via NF-κB and BCL-2/BAX modulation in isoproterenol-induced myocardial injury

**DOI:** 10.3389/fphar.2025.1628715

**Published:** 2025-10-14

**Authors:** Fayyaz Anjum, Saad Touqeer, QurratUlAin Jamil, Ayesha Rida, Esraa M. Haji, Sulaiman Mohammed Abdullah Alnasser, Ali F. Almutairy, Hajar Alghamdi, Ashfaq Ahmad, Shahid Muhammad Iqbal

**Affiliations:** ^1^ Department of Pharmacology, Faculty of Pharmacy, The Islamia University of Bahawalpur, Bahawalpur, Pakistan; ^2^ College of Pharmacy, Al Ain University, Abu Dhabi, United Arab Emirates; ^3^ AAU Health and Biomedical Research Center, Al Ain University, Abu Dhabi, United Arab Emirates; ^4^ Department of Pharmacy Practice, Faculty of Pharmacy, The Islamia University of Bahawalpur, Bahawalpur, Pakistan; ^5^ Department of Pharmaceutical Chemistry, College of Pharmacy, University of Hafr Al Batin, Hafr AlBatin, Saudi Arabia; ^6^ Department of Pharmacology and Toxicology, College of Pharmacy, Qassim University, Buraydah, Saudi Arabia; ^7^ Department of Pharmacy Practice, College of Pharmacy, University of Hafr Al Batin, Hafr AlBatin, Saudi Arabia; ^8^ Michael Sars Center, University of Bergen, Bergen, Norway

**Keywords:** *Bassia indica*, myocardial infarction, inflammation, apoptosis, anti-inflammatory

## Abstract

**Background:**

Myocardial infarction (MI) is a fatal coronary heart disease that develops due to prolonged hypoxia. During MI progression, uncontrolled inflammation and apoptosis mediated by NF-κB and BAX/BCL-2 signaling pathways potentiate cardiac injury. Phenolic and flavonoid-rich medicinal plants have shown efficacy in suppressing the inflammatory pathways, thus reducing the adverse cardiac remodeling. In this study, we evaluated the cardioprotective and anti-inflammatory potential of *Bassia indica* (Wight) A.J. Scott, a plant traditionally used for treating cardiac disorders.

**Methods:**

*B. indica* extract (BiE) was prepared and characterized by UHPLC-MS/MS. Its anti-inflammatory activity was determined by *in vitro* inhibition of COX-2 and 5-LOX enzymes, followed by *in vivo* suppression of acute inflammation induced by carrageenan, histamine, and serotonin. The role of the anti-inflammatory activity in the amelioration of myocardial injury was assessed by isoproterenol (ISO)-induced MI, and qPCR studies were performed to explore underlying mechanisms.

**Results:**

UHPLC/MS/MS analysis of BiE tentatively identified several plant metabolites, including kaempferol 3-glucoside-7-sophoroside, kaempferol 3-rutinoside-7-sophoroside, and kaempferol 3-(2G-glucosylrutinoside), and phenolic derivatives. It inhibited COX-2 (IC_50_ = 0.6 μg/mL) and 5-LOX (IC_50_ = 8.3 μg/mL) enzymes. BiE-treated animals exhibited reduced inflammation in response to carrageenan, histamine, and serotonin. Pretreatment with BiE significantly reduced the infarct size; preserved cardiac tissue architecture; lowered cardiac biomarkers (cTnI, CK-MB, LDH, and AST); downregulated NF-κB, COX-2, TNF-α, and IL-1β; and upregulated IL-10 and BCL-2.

**Conclusion:**

These findings suggest that BiE has cardioprotective effects that are mediated by the suppression of inflammation and apoptosis.

## Graphical Abstract

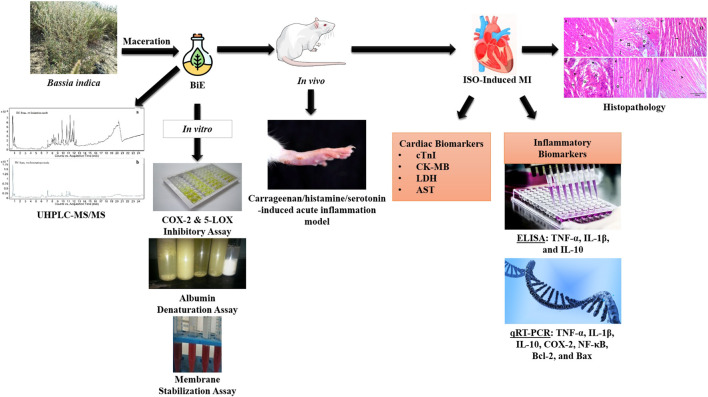

## 1 Introduction

Cardiovascular diseases (CVDs) are increasing globally due to sedentary lifestyles and account for nearly one-third of all reported deaths. Despite extensive research on CVDs, ischemic heart disorders remain a challenge and pose a serious threat to healthy life. Myocardial infarction (MI) develops from persistent ischemia caused by an imbalance between the oxygen demand and supply (Kaier et al., 2021). Ischemia is not the sole factor in MI development; secondary pathological processes such as oxidative stress and inflammation are also interconnected and form a complex signaling network that ultimately leads to cardiac cell injury and apoptosis (Duan et al., 2023). Many patients diagnosed with acute myocardial infarction (AMI) show signs of increased inflammation, which appear in two (pro-inflammatory and anti-inflammatory) phases. The first phase of inflammation that starts after AMI involves the activation of transcription factor NF-κB, which subsequently activates several processes such as the complement cascade, generation of reactive oxygen species (ROS), and the expression of pro-inflammatory cytokines (TNF-α, IL-1β, IL-6, and IL-18), COX-2, and antiapoptotic gene BCL2 (Olsen et al., 2022). Controlled inflammation is necessary to clear dead cells and heal myocytes; however, if this inflammatory response becomes uncontrolled and expands excessively, it can lead to adverse myocardial remodeling. The second anti-inflammatory reparative phase suppresses and resolves the pro-inflammatory phase by modifying the functions of infiltrating leukocytes, thereby subduing the inflammation (Zhang and Dhalla, 2024). The transformation between these two stages is managed by a highly modulated and composite interaction between different cells in the heart (including cardiomyocytes, fibroblasts, interstitium, and endothelial cells) and components of the immune system (including lymphocytes, macrophages, neutrophils, monocytes, and dendritic cells). Any change in balance or transformation between these two phases can enhance the myocardial trauma and post-MI adverse left ventricular remodeling (Ong et al., 2018).

Recognizing inflammation as a key factor in the development of AMI and the involvement of NF-κB in augmenting inflammation, anti-inflammatory interventions that target NF-κB are gaining attention as a therapeutic strategy to manage MI. Recent clinical trials have confirmed that anti-inflammatory therapies such as canakinumab (IL-1β inhibitor) and granulocyte colony-stimulating factor prevent left ventricular remodeling after MI and improve cardiac functions (Viola et al., 2021). Commercially available steroidal and nonsteroidal anti-inflammatory drugs have also been applied clinically to treat MI. However, they failed to arrest the cardiac damage effectively due to their inability to counter the multiple etiologies involved in the progression of cardiac injury (Liu et al., 2024). Furthermore, increased incidences of cardiac rupture were observed with corticosteroid treatment, which overcame its beneficial anti-inflammatory effects in MI. NSAIDs are also not advised in MI as a higher risk of bleeding and thrombotic events were observed (Dewenter et al., 2016). Considering the limitations of the available treatments, there is a need to explore new agents with alternative and novel approaches for cardiac protection. The isoproterenol (ISO)-induced MI model is a frequently used experimental model to investigate new drugs for the treatment of myocardial injury. ISO is a β-receptor agonist that, at supramaximal doses in experimental animals, causes myocardial stress and induces infarction that is similar to human acute MI. Auto-oxidation of ISO produces free radicals, ROS, lipid peroxidation, and Ca^2+^ overload during cardiac injury, which leads to inflammation, cell necrosis, and apoptosis (Zhang et al., 2023). Amid the pursuit of alternative therapeutic strategies, interventions targeting the downregulation of the NF-κB signaling pathway have been observed to be beneficial in ameliorating myocardial damage. The identification of this new target paved the way for further research, and numerous studies have reported that phenolic compounds present in medicinal plants can mitigate cardiac injury by suppressing NF-κB and COX-2 (Anajirih et al., 2024; Verma et al., 2019).

*Bassia indica* (Wight) A.J. Scott (synonyms: *Kochia indica*) is a well-known, locally used medicinal plant that is used as a cardiotonic and diuretic agent to treat cardiac ailments by local communities in countries such as Pakistan (Niaz et al., 2013; Umair et al., 2017), India (Tripathi et al., 1996), Saudi Arabia (Youssef, 2013), and Tunisia (Bouaziz et al., 2009). Other plants of the genus *Bassia*, such as *Bassia scoparia*, known as Di-Fu-Zi, are valued in traditional Chinese medicine and used as a cardiotonic (Grabowska et al., 2023). *B. indica* has been documented to have therapeutically active phytochemicals and anti-inflammatory activities, but its role in cardiac protection has not been elucidated (Othman et al., 2022). In this context, the current study was designed to investigate the anti-inflammatory and cardioprotective potential of *B. indica* and to explore the mechanisms underlying its effects.

## 2 Materials and methods

### 2.1 Plant collection, extraction, and fractionation

Before the flowering stage, the *B. indica* plant (10 kg) was harvested by a field expert from Muzaffargarh, a southern district of Punjab, Pakistan [31°10′5″N 70°50′25″E]. A botanist identified the plant, and a voucher number (324/Botany) was obtained after the specimen was submitted. The powdered plant material was extracted with 70% methanol by maceration for 7 days. Then, it was filtered and concentrated using a rotary evaporator to obtain a semi-solid *B. indica* crude extract (BiE) (Iqbal et al., 2016). A portion of the extract was used for fractionation, and n-hexane, dichloromethane, ethyl acetate, n-butanol, and aqueous fractions were prepared as described in our previous work (Anjum et al., 2024).

### 2.2 UHPLC-MS/MS

For UHPLC-MS/MS analysis of BiE, two solvents, that is, 0.1% CH_2_O_2_ in acetonitrile (solvent A) and 0.1% CH_2_O_2_ in deionized water (solvent B), were prepared. A gradient (5%–100%) was eluted for 30 min at the flow rate of 0.5 mL/min, and 1 μL of the BiE sample was used. The conditions of MS operations are provided in the supplementary data. The Metlin AM PCDL-N-170502 search database was used for identifying the metabolites (Aslam et al., 2024).

### 2.3 *In vitro* anti-inflammatory assays

#### 2.3.1 Cyclooxygenase inhibitory assay

A 10 µL of activated COX-2 enzyme solution was mixed with 50 µL cofactor solution. Then, 20 μL of BiE or celecoxib concentrations (7.81 μg/mL–1,000 μg/mL) was added and incubated for 5–10 min. After incubation, 20 μL of 30 mM arachidonic acid solution was added to the mixture to initiate the reaction, and the mixture was incubated at 37 °C for 15 min. At the end, the reaction was stopped by adding HCl, and the absorbance was measured at 570 nm (Aslam et al., 2024).

#### 2.3.2 Lipoxygenase inhibitory assay

LOX inhibition potential was measured by using human recombinant 5-lipoxygenase. A 250-µL enzyme solution (10,000 U/mL) and 250 μL of different concentrations of BiE (7.81 μg/mL–1,000 μg/mL) or montelukast sodium were mixed and incubated for 10 min. Then, 1 mL of 0.6 mM linoleic acid was added to the mixture, and the absorbance was measured at 234 nm (Aslam et al., 2024).

#### 2.3.3 Protein denaturation inhibition

Fresh egg albumin was mixed with phosphate buffer (pH 6.4) and Tween 80 (1% of toal voulme was added). Reaction mixtures were prepared by adding 3 mL of freshly prepared egg albumin and 2 mL of different BiE/diclofenac sodium solutions (10–1,000 μg/mL). After 15 min of incubation at 37 ± 2 °C, the solutions were kept at 70 °C for 5 min. Absorbance was measured at 660 nm after the mixtures were cooled to room temperature, using distilled water as the control. The following equation was used to calculate the % protein denaturation inhibition (Nawaz et al., 2023).

$$\%\text{ inhibition}=\left(\frac{\text{Abs}.\;\text{of}\;\text{control}-\text{Abs}.\;\text{of}\;\text{sample}}{\text{Abs}.\;\text{of}\;\text{control}}\right)\times 100.$$

#### 2.3.4 Membrane stabilizing test

Equal volumes of blood and sterile Alsever’s solution were mixed and centrifuged at 3,000 rpm for 10 min to obtain packed red blood cells (RBCs). RBCs were separated and washed three times with the same volume of isotonic buffered saline (pH 7.4) and centrifuged at 1,000 rpm. A 10% (v/v) RBC suspension was prepared in isotonic phosphate buffer saline and stored at 4 °C. In a test tube, 0.5 mL of BiE/diclofenac sodium solutions (10–1,000 μg/mL) was added along with 2 mL of hypotonic saline (0.45% NaCl). Afterward, a 0.5 mL RBC suspension was added. The control reaction contained only hypotonic saline and a RBC suspension. The reaction mixtures were kept at 37 °C for 30 min and then centrifuged at 3,000 rpm for 10 min to obtain supernatants. The absorbance of the supernatants was measured at 560 nm to calculate the percent inhibition (Nawaz et al., 2023).

$$\%\text{ inhibition}=\left(\frac{\text{Abs}.\;\text{of}\;\text{control}-\text{Abs}.\;\text{of}\;\text{sample}}{\text{Abs}.\;\text{of}\;\text{control}}\right)\times 100.$$

### 2.4 *In vivo* studies

#### 2.4.1 Experimental animals

Animals were housed under standard laboratory conditions, as described previously (Masood et al., 2023). Before experimentation, the animals were acclimatized for 2 weeks, and the study was approved by the Pharmacy Animal Ethics Committee (PAEC file no PAEC/23/101).

#### 2.4.2 Carrageenan/histamine/serotonin-induced acute inflammation

Normal Wistar rats (150–200 g) were randomly divided into six groups and fasted for 18 h before the study. After weighing, the left hind paw size (0 h) of all the animals was measured. The normal and diseased groups received normal saline (5 mL/kg), whereas the treatment groups were given 30, 100, and 300 mg/kg BiE orally. The standard group was given piroxicam/loratadine 10 mg/kg or cyproheptadine 5 mg/kg orally. After 1 h of pretreatment, 100 µL of 1% carrageenan/histamine/serotonin was administered into the subplantar region of the left hind paw of each rat, except the normal group. Rats were kept separately, and the paw size was measured every hour up to 6 h. The percentage of inflammation inhibition was determined using the following formula (Nawaz et al., 2023):

$$\%\text{ inhibition of inflammation}=\left(\frac{\text{Increase}\;\text{in}\;\text{paw}\;\text{size}\;\left(\text{diseased}\right)-\text{Increase}\;\text{in}\;\text{paw}\;\text{size}\;\left(\text{sample}\right)}{\text{Increase}\;\text{in}\;\text{paw}\;\text{size}\;\left(\text{diseased}\right)}\right)\times 100.$$

#### 2.4.3 Isoproterenol-induced myocardial infarction

Healthy male Wistar rats (180–250 g) were weighed and divided into six groups. Normal and ISO (diseased) groups received NS (4 mL/kg), whereas 30, 100, and 300 mg/kg of BiE was administered to the respective treatment groups. Carvedilol (10 mg/kg) was administered to the standard group. All treatments were given orally for 26 days. On the 25th and 26th days, ISO (150 mg/kg) was administered subcutaneously to all groups, except the normal group. After 12 h of the second ISO dose, animals were anesthetized with xylazine and ketamine (1:10 ratio), blood samples were obtained, and sera were separated. Dissected hearts were washed with ice-chilled NS, weighed, and divided into three parts. One part was used for triphenyl tetrazolium chloride (TTC) staining, whereas the second part was stored at −20 °C and used for assessing the mRNA levels of TNF-α, IL-1β, IL-10, COX-2, NF-κB, BAX, and BCL-2 genes. The third portion was kept in 10% buffered formalin solution for histopathological studies. Collected sera were stored at −20 °C and used for the measurement of cardiac markers (cTnI, CKMB, LDH, and AST) (Gupta et al., 2024; Ibrar et al., 2019).

##### 2.4.3.1 Myocardial infarct size measurement

Infarct size was measured with 2,3,5 triphenyl tetrazolium chloride (TTC) staining according to the described method, with some modifications (Liu et al., 2021). After dissection, heart tissue was washed with PBS, wrapped in a clean plastic sheet, and frozen at −20 °C for 1 h to harden the tissue. The frozen heart tissue was then sliced transversely into 2–3 mm sections with a sharp surgical blade from the middle of the heart for better exposure of the left ventricle. The sections were then stained with a TTC solution (1% in PBS) for 20 min at 37 °C with occasional shaking, followed by fixation with 10% formalin-buffered saline for 30 min. Non-infarcted heart tissue was stained brick-red, whereas the infarcted area appeared pale white. After staining, sections were photographed, the pictures were analyzed using ImageJ software, and the infarct size was calculated.

##### 2.4.3.2 Assessment of cardiac markers

Cardiac marker cTnI was assessed in the serum with an ELISA kit according to the manufacturer’s (Nanjing Pars Biochemical) protocol, whereas CKMB, LDH, and AST were measured in the serum with commercially available colorimetric kits according to the manufacturer’s (Bio-Science Medical, Spain) protocol with Microlab 300 (Merck, Germany).

##### 2.4.3.3 Total protein content and inflammatory markers assessment

The total protein content in the heart tissue homogenate was measured using the Bradford method. Inflammatory markers TNF-α, IL-1β, and IL-10 were assessed in the serum with ELISA kits according to the manufacturer’s (Nanjing Pars Biochemical) protocol. Absorbance was measured at 450 nm with a BioTek Synergy HT microplate reader.

##### 2.4.3.4 qPCR studies

The expression of TNF-α, IL-1β, IL-10, COX-2, NF-κB, BCL-2, and BAX genes was quantified using real-time PCR following a previously used method (Fiaz et al., 2024). Briefly, total RNA was isolated from cardiac tissue with the HiPure Total RNA kit, and complementary DNA (cDNA) was synthesized using the Thermo Fisher cDNA synthesis kit. qPCR was performed on a SLAN-96P real-time PCR system, whereas Ct values were analyzed using SLAN-96P software. Relative expression levels were normalized against the housekeeping gene (GAPDH). The primer sequences used in this study are listed in Supplementary Table S1.

##### 2.4.3.5 Histological analysis of heart tissues

The heart tissues of the animals in each group were excised and stored in 10% formalin for fixation. The tissues were washed in running water for 8–12 h to remove the fixative agent and then dehydrated with different concentrations, that is, 70% (3 h), 80% (1 h), 95% (2 h), and 100% (3 h) ethanol. After that, ethanol was removed by placing the tissues in absolute xylene for 3 h, and then they were embedded in paraffin wax and cooled at 4 °C overnight. Then, tissue sections (5–10 µm thick) were cut using a microtome and stained with hematoxylin and eosin (H&E). Stained tissues were observed under a camera-fitted microscope at 40X, and pictures were obtained with PixelPro software, followed by characterization for inflamed cardiac tissues, neutrophil infiltration, edema, and any other injury (Aslam et al., 2024).

### 2.5 Statistical analysis

GraphPad Prism (version 10) was used for analysis. The results were expressed as the mean ± SEM, and depending upon suitability, one-way or two-way ANOVA was applied, followed by Tukey’s multiple comparison test for significance calculation; *p-*value *<* 0.05 was considered statistically significant.

## 3 Results

### 3.1 BiE preparation and fractionation

An amount of 264 g of BiE was obtained after extraction with a percentage yield of 8.8%. Fractionation results showed that an aqueous fraction had the highest yield (10.5%), followed by n-butanol (3%), ethyl acetate (2%), dichloromethane (1.5%), and *n*-hexane fractions (1%).

### 3.2 UHPLC-MS/MS analysis of BiE

UHPLC-MS/MS analysis of BiE tentatively identified several plant-derived metabolites in the negative and positive modes. These metabolites include flavonoid glycosides such as kaempferol 3-glucoside-7-sophoroside, kaempferol 3-rutinoside-7-sophoroside, and kaempferol 3-(2G-glucosylrutinoside), along with phenolic acids. Tentative identifications were based on spectral matching with the METLIN database. The identified metabolites found in BiE are listed in Table 1, whereas speculative metabolites are listed in Supplementary Table S2. The mass spectra of all the identified compounds, total ion chromatogram (TIC), and total compound chromatogram (TTC) are shown in Supplementary Figures S1, S2.

**TABLE 1:** Tentatively identified BiE metabolites through UHPLC-MS/MS in the negative and positive modes.

Sr. no	RT (min)	Metabolites	MF	MW (g/mol)	Base peak (m/z)	Previous occurrence
1	7.62	7-Epi-12-hydroxyjasmonic acid glucoside	C_18_H_28_O_9_	388.4	387.1	Dussarrat et al. (2022)
2	8.13	Kaempferol 3-glucoside-7-sophoroside	C_33_H_40_O_21_	772.7	771.2	Malik et al. (2023)
3	8.37	Kaempferol 3-rutinoside-7-sophoroside	C_39_H_50_O_25_	918.8	917.2	Pillanjinayya et al. (2025)
4	8.39	Isorhamnetin 3-glucosyl-(1->2)-[rhamnosyl-(1->6)-galactoside]	C_34_H_42_O_21_	786.7	785.2	Chauhan et al. (2024)
5	8.39	Kaempferol 3-(2G-glucosylrutinoside)	C_33_H_40_O_20_	756.7	755.2	Arshad et al. (2021)
6	8.79	Robinetin 3-rutinoside	C_27_H_30_O_16_	610.5	609.1	Pervaiz et al. (2022)
7	9.11	(3S,7S)-Jasmonic acid	C_12_H_18_O_3_	210.2	245.0	Vick and Zimmerman (1984)
8	9.12	Luteolin 7-rhamnosyl (1->6) galactoside	C_27_H_30_O_15_	594.5	593.1	Pervaiz et al. (2022)
9	9.18	Tricetin 7-methyl ether 3′-glucoside-5′-rhamnoside	C_28_H_32_O_16_	624.5	623.1	Arshad et al. (2021)
10	10.00	Ferulic acid	C_10_H_10_O_4_	194.1	193.0	Kumar and Pruthi (2014)
11	11.11	Pyropheophorbide a	C_33_H_34_N_4_O_3_	534.6	533.2	Saide et al. (2020)
12	11.62	6-Feruloylglucose 2,3,4-trihydroxy-3-methylbutylglycoside	C_21_H_30_O_12_	474.5	473.1	Mopai et al. (2023)

### 3.3 Anti-inflammatory activity of BiE

#### 3.3.1 COX-2 inhibitory assay

The COX-2 inhibition potential of BiE and its fractions was determined and compared with those of celecoxib. The results showed that BiE has the maximum COX-2 inhibitory potential, with a calculated IC_50_ of 0.6 μg/mL, which is comparable to that of celecoxib (0.2 μg/mL). The IC_50_ values of other fractions were as follows: ethyl acetate (3.2 μg/mL), *n*-hexane (3.4 μg/mL), dichloromethane (11.5 μg/mL), aqueous (18.5 μg/mL), and n-butanol (40.2 μg/mL).

#### 3.3.2 5-LOX inhibitory assay

The results showed that the ethyl acetate fraction has the maximum 5-LOX inhibitory effect with an IC_50_ of 5.1 μg/mL. Similarly, BiE also showed good 5-LOX enzyme inhibition with an IC_50_ value of 8.3 μg/mL. The highest IC_50_ was observed in the n-butanol fraction (31.0 μg/mL), followed by dichloromethane (17.8 μg/mL), aqueous (9.1 μg/mL), and *n*-hexane (8.1 μg/mL) fractions. The LOX IC_50_ value for montelukast was 1.2 μg/mL.

#### 3.3.3 Protein denaturation and membrane stabilization activity

The *in vitro* anti-inflammatory effects of BiE were assessed using albumin denaturation and RBC membrane stabilization assays. Percent albumin protein denaturation and RBC hemolysis inhibition by BiE were observed in a dose-dependent manner, with the maximum inhibition of 71.9% ± 3.4% and 80.7% ± 2.0%, respectively, at a dose of 1 mg/kg. The IC_50_ value for BiE protein denaturation inhibition was 273 μg/mL, and for diclofenac, it was 227 μg/mL. The IC_50_ of BiE for RBC membrane stabilization was 99.84 μg/mL, and for diclofenac, it was 66.34 μg/mL. All the results were comparable to those of the standard drug, diclofenac sodium, and no significant difference was observed.

#### 3.3.4 Carrageenan-induced paw edema

**FIGURE 1:**
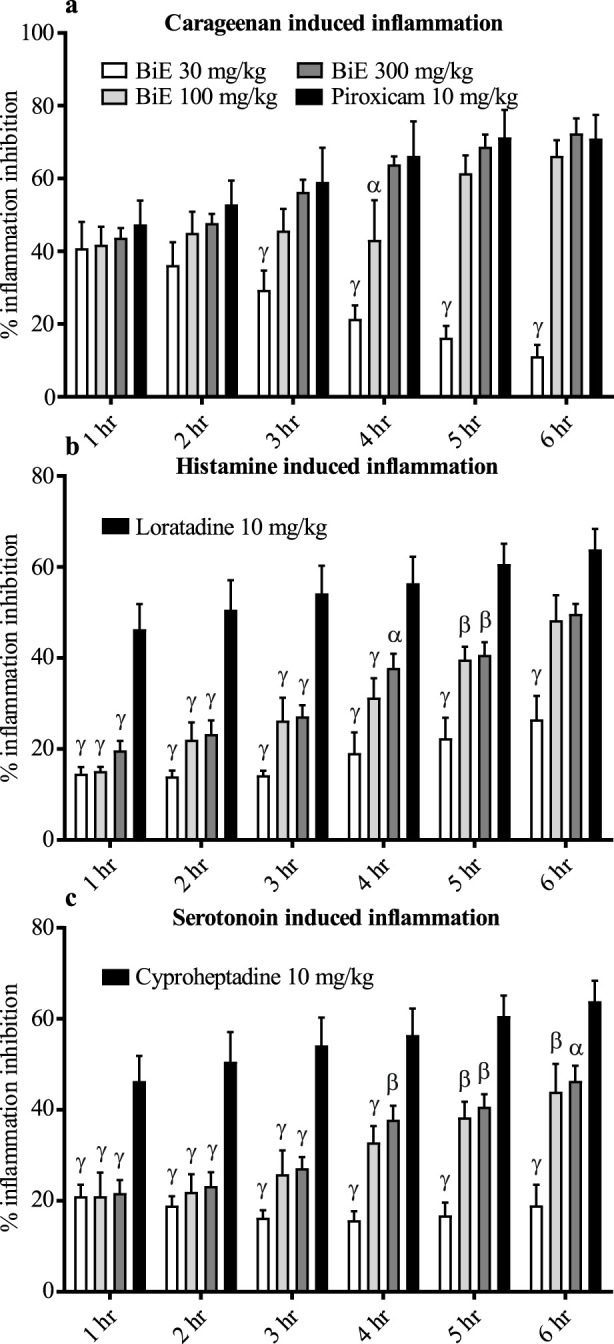
Anti-inflammatory effect of BiE on **(a)** carrageenan-, **(b)** histamine-, and **(c)** serotonin-induced inflammation. Data are presented as the mean ± SEM. Two-way ANOVA followed by Tukey’s test was applied to determine significance among the groups, and BiE effects were compared with those of **(a)** piroxicam, **(b)** loratadine, and **(c)** cyproheptadine. Significance is denoted as α = *p* < 0.05, β = *p* < 0.01, and γ = *p* *<* 0.001.

The effect of BiE on the percent inflammation inhibition of carrageenan-induced paw edema was dose-dependent, with the maximum percent inflammation inhibition of 72.4% ± 4.1% observed at 300 mg/kg at 6 h. The effect of BiE 300 mg/kg was comparable to that of piroxicam. BiE 100 mg/kg showed 66.2% ± 4.3% inhibition of inflammation at 6 h. Inflammation inhibition in BiE 30 mg/kg-treated animals decreased over time, with values of 40.87% ± 7.20% at the first hr and 11.22% ± 3.01% at the sixth hr (Figure 1a).

#### 3.3.5 Histamine-induced paw edema

BiE inhibited the histamine-induced inflammation, and its anti-inflammatory effect increased with time. The maximum effect was observed in the 300 mg/kg-treated group at 6 h, with 49.7% ± 2.2% inflammation inhibition, which was 48.3% ± 5.8% and 26.5% ± 5.2% in the 100 and 30 mg/kg-treated groups at the same time, respectively. Loratadine (5 mg/kg) showed superior response until 5 h, but at 6 h, the maximum inhibition was 63.9% ± 4.5%, which was comparable to that of BiE 100 and 300 mg/kg (Figure 1B).

#### 3.3.6 Serotonin-induced paw edema

BiE treatment also relieved serotonin-induced inflammation, but cyproheptadine (10 mg/kg) showed a superior response at all time-points. The maximum percent inflammation inhibition observed was 18.9 ± 4.5, 43.9 ± 6.1, and 46.4 ± 3.3% at the doses of 30, 100, and 300 mg/kg, respectively, whereas cyproheptadine inhibited 63.8 ± 4.5% inflammation (Figure 1c).

### 3.4 Cardioprotective activity of BiE

MI was successfully induced by subcutaneous administration of two successive doses of ISO (150 mg/kg). The ISO dose was selected through a pilot study, and two animals died during the study.

#### 3.4.1 Effect on the infarct size

**FIGURE 2:**
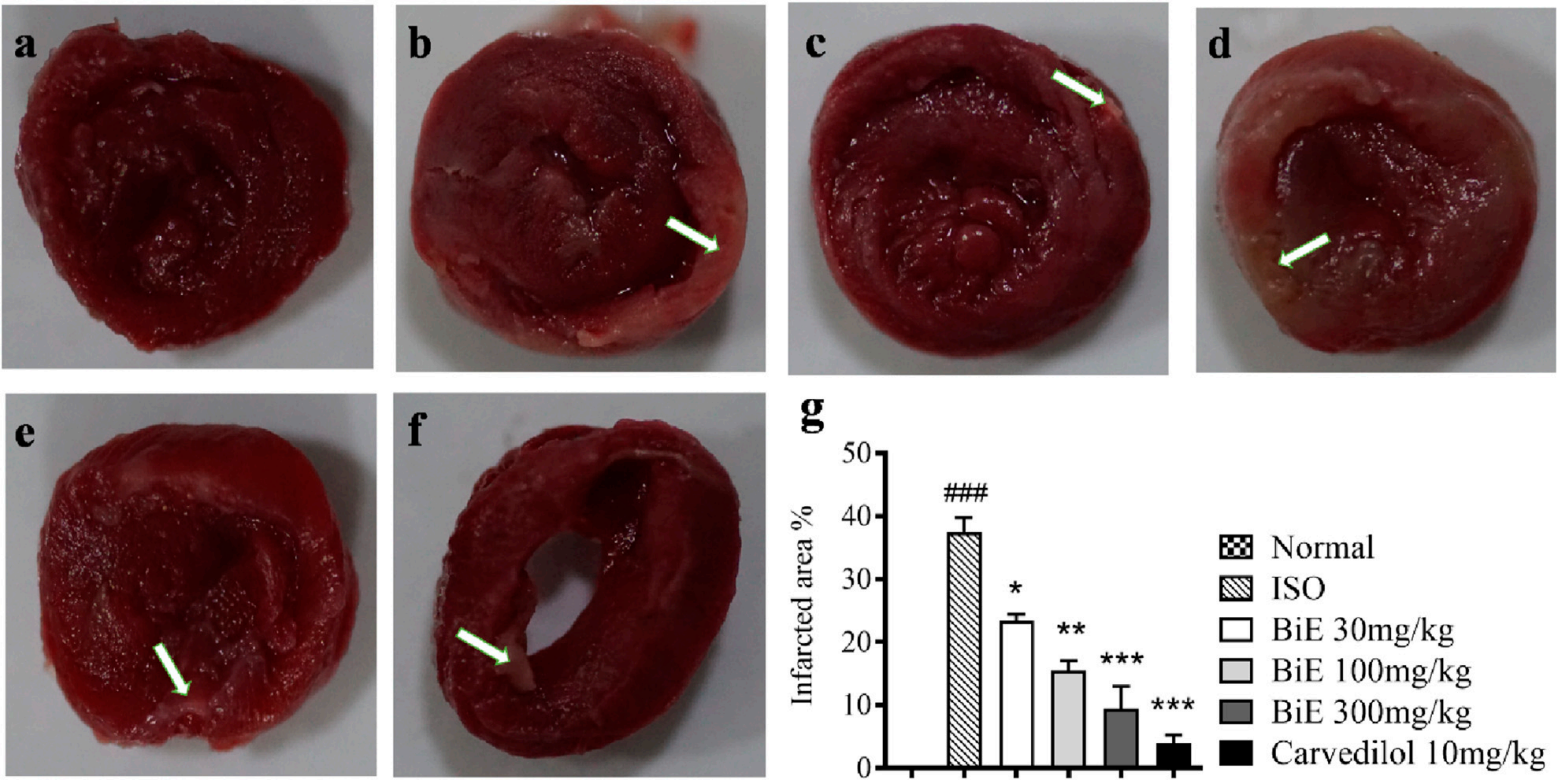
Effect of BiE and carvedilol (10 mg/kg) along with the normal and ISO groups on heart tissue observed with TTC staining in ISO-induced MI (n = 3). **(a)** Normal, **(b)** ISO, **(c)** carvedilol, **(d)** BiE 30, **(e)** BiE 100, **(f)** BiE 300 mg/kg, and **(g)** infarct quantification. Brick-red-stained areas show viable myocardium, whereas white or unstained areas (indicated by the white arrow) represent infarcted areas. The ISO group was compared with the control group, and significance is indicated as follows: ### = *p* < 0.001. In contrast, BiE and carvedilol were compared with the ISO group, and significance is denoted as follows: * = *p* < 0.05, ** = *p* < 0.01, and *** = *p* < 0.001.

TTC stained the viable myocardium a brick-red color, whereas the infarcted areas remained white, pale tan, or unstained, as shown in Figure 2. White patches or unstained areas are directly proportional to cardiac injury, and large infarcted areas (stained tissue) were observed in the ISO group (Figure 2b). Pretreatment with different doses of BiE decreased the infarct size dose-dependently, as clearly seen in Figures 2c–f. The infarct size was quantified by ImageJ software; the percentage of the infarcted area was seen to be the highest in the ISO group (37 ± 2.3%), and it was significantly (*p* < 0.001) higher than that in the normal group. A significant decrease in the percent infarct area, 23 ± 0.97, 16 ± 1.5, and 9.4 ± 3.6%, was observed as the dose of BiE increased to 30, 100, and 300 mg/kg, respectively. The lowest infarct area (3.9 ± 1.3%) was observed in the carvedilol 10 mg/kg-treated group (Figure 2g).

#### 3.4.2 Cardiac biomarkers

**FIGURE 3:**
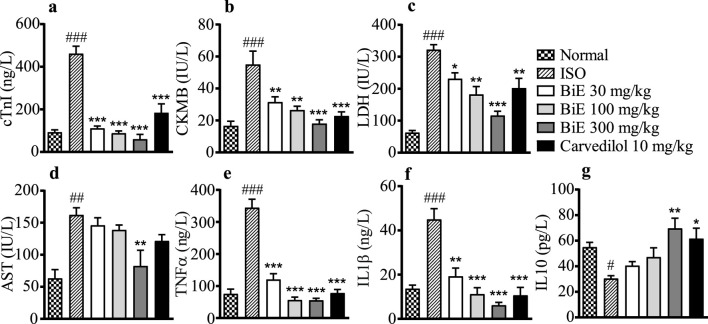
Effect of BiE and carvedilol on serum levels of **(a)** cTnI, **(b)** CKMB, **(c)** LDH, **(d)** AST, **(e)** TNF-α, **(f)** IL-1β, and **(g)** IL-10 in ISO-induced MI. Data is presented as the mean ± SEM (n = 3). One-way ANOVA followed by Tukey’s test was used to analyze the results. The ISO group was compared with the control group, and significance is indicated as follows: # = *p* < 0.05, ## = *p* < 0.01, and ### = *p* < 0.001. In contrast, BiE and carvedilol were compared with the ISO group, and significance is denoted as follows: * = *p* < 0.05, ** = *p* < 0.01, and *** = *p* < 0.001.

We observed elevated cTnI in the ISO group (461 ± 35 ng/L) as compared to the normal control group (93 ± 11 ng/L), indicating cardiac tissue damage. cTnI levels were decreased in treatment groups, with the maximum effect observed in BiE 300 mg/kg (60 ± 22 ng/L, Figure 3a). BiE also decreased (*p* < 0.001) CKMB, LDH, and AST levels, with the maximum reduction (18 ± 2.4 IU/L, 116 ± 13 IU/L, and 83 ± 25 U/L, respectively) at 300 mg/kg in comparison to the ISO group, and the results were comparable to that of carvedilol (Figures 3b–d). The decrease in the levels of these cardiac markers with BiE treatment at 30 and 100 mg/kg was also significant (*p* < 0.01), except for the AST levels.

#### 3.4.3 Effect on interleukins

The ISO group showed increased pro-inflammatory markers, TNF-α (345 ± 26 ng/L) and IL-1β (45 ± 4.9 ng/L), whereas a decrease in the anti-inflammatory marker IL-10 (30 ± 2.2 pg/L) was observed in the ISO group compared to the normal control group. BiE treatment improved the inflammatory markers, with the maximum response observed at 300 mg/kg, as TNF-α and IL-1β levels were reduced (*p* < 0.01) compared to those in the ISO group. BiE 300 mg/kg also increased the anti-inflammatory marker IL-10 (70 ± 8.0 pg/L) compared to that in the ISO group. The results of the carvedilol (10 mg/kg) group were comparable to those of the BiE 300 mg/kg group (Figures 3d–f).

#### 3.4.4 Effect on mRNA levels of inflammatory markers

**FIGURE 4:**
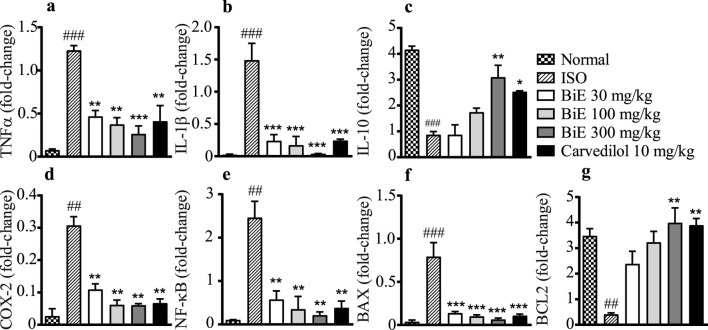
Effects of BiE and carvedilol on mRNA levels of **(a)** TNF-α, **(b)** IL-1β, **(c)** IL-10, **(d)** COX-2, **(e)** NF-κB, **(f)** BAX, and **(g)** BCL-2 in isoproterenol-induced MI. Data is presented as the mean ± SEM (n = 3). One-way ANOVA followed by Tukey’s test was used to analyze the results. The ISO group was compared with the control group, and significance is indicated as ## = *p* < 0.01 and ### = *p* < 0.001. In contrast, BiE and carvedilol were compared with the ISO group, and significance is denoted as follows: * = *p* < 0.05, ** = *p* < 0.01, and *** = *p* < 0.001.

BiE effects on the mRNA expression of inflammatory interleukins, including TNF-α, IL-1β, and IL-10, are presented in Figures 4a–c. BiE decreased (*p* < 0.01) the mRNA levels of pro-inflammatory markers, that is, TNF-α and IL-1β, while increased the mRNA levels of anti-inflammatory interleukin IL-10 at all three doses (30, 100, and 300 mg/kg) compared to that in the ISO group. Carvedilol 10 mg/kg also decreased (*p* < 0.05) the expression of TNF-α and IL-1β while increased the expression of IL-10. The results of BiE were comparable to those of carvedilol.

#### 3.4.5 Effect on COX-2 and NF-κB mRNA

The ISO group showed a significant increase in NF-κB (2.5 ± 0.34-fold change, Figure 4d) and COX-2 (0.31 ± 0.03-fold change, Figure 4e) compared to the normal control group. Groups treated with BiE showed significant decrease in NF-κB and COX-2 levels, and the maximum response was observed with BiE 300 mg/kg (0.21 ± 0.08 and 0.060 ± 0.005-fold change, respectively) followed by BiE 100 mg/kg (0.35 ± 0.29 and 0.061 ± 0.02, respectively) and BiE 30 mg/kg (0.57 ± 0.20 and 0.11 ± 0.02, respectively) compared to the ISO group. Similarly, carvedilol significantly decreased the levels of NF-κB (2.5 ± 0.37) and COX-2 (0.31 ± 0.03).

#### 3.4.6 Effect on BAX and BCL-2 mRNA

ISO increased the BAX protein expression in the ISO group (0.79 ± 0.16-fold change) and decreased the levels of BCL-2 (0.41 ± 0.06-fold change) compared to the normal group (0.035 ± 0.022 and 3.5 ± 0.3-fold change, respectively). BiE treatment ameliorated the ISO effects on the BAX protein and decreased its levels (*p* < 0.001). The observed levels for BAX were 0.14 ± 0.02, 0.099 ± 0.02, and 0.063 ± 0.017-fold change at the doses of 30, 100, and 300 mg/kg BiE, respectively. The BiE results were comparable to those of carvedilol 10 mg/kg (0.11 ± 0.018-fold change, Figure 4f). BiE increased BCL-2 mRNA levels by 2.4 ± 0.5, 3.2 ± 0.43, and 4.0 ± 0.59-fold at 30, 100, and 300 mg/kg, respectively, in contrast to that in the ISO group. An increase in the BCL-2 gene expression at BiE 300 mg/kg was significant, whereas no significant increase was observed at lower doses (30 and 100 mg/kg). Carvedilol also increased (3.9 ± 0.26-fold change) compared to the ISO group (Figure 4g).

#### 3.4.7 Effect on cardiac tissue architecture

**FIGURE 5:**
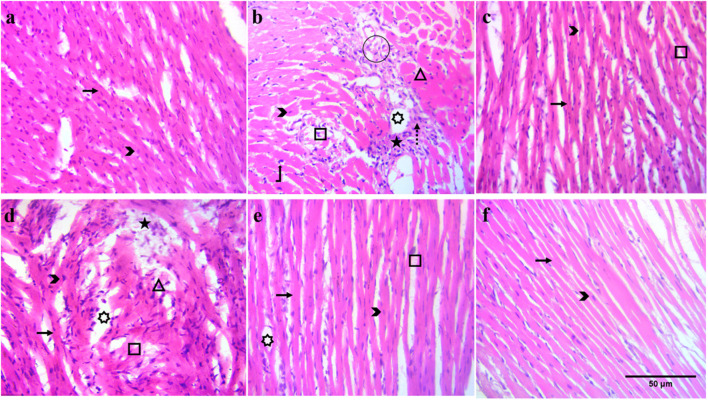
Effect of BiE and carvedilol along with the normal and ISO groups on heart histopathological alterations at 40X (scale bar: 50 µm) in ISO-induced MI (n = 3). **(a)** Normal, **(b)** ISO, **(c)** carvedilol 10 mg/kg, **(d)** 30 mg/kg, **(e)** 100 mg/kg, and **(f)** 300 mg/kg. Signs indicate the following: arrow: normal cardiac fibers, arrowhead: myocardial nuclei, square: broken cardiac fibers, star: neutrophil infiltrate, rectangle: inflamed cardiac myocytes, circle: tissue edema, dashed arrow: collagen deposition, bent-up arrow: hyperchromatic nuclei, and 7-point star: intracellular vacuolation of cardiomyocytes.

Marked histopathological alterations, including neutrophil infiltration, inflamed myocytes, and broken muscles, were observed in the ISO group (Figure 5b), along with a decreased muscular content compared to the normal group. The number of nuclei was lower, and multiple cells were found without a nucleus. Edema was also observed at several positions in the ISO group tissues. At all doses (30, 100, and 300 mg/kg), BiE and carvedilol (10 mg/kg) resisted ISO-induced tissue injury, and reduced tissue alterations were observed (Figures 5c–f).

## 4 Discussion

*B. indica* is traditionally used for cardiac ailments and has shown good anti-inflammatory activity in both *in vitro* and *in vivo* assays. It mitigated stress-induced cardiac injury by restoring cardiac tissue architecture, improving cardiac injury markers, and reducing the levels of inflammatory mediators. Inflammation is a complex, normal defensive response to cellular injury caused by physical or chemical stimuli. Controlled inflammatory response is a key to removing injurious stimuli and restoring normal physiological conditions. However, if hemostatic control of inflammation is lost, this reparative physiological response can worsen many diseases, including MI. Numerous studies have demonstrated that medicinal plants can help arrest uncontrolled inflammation and prevent inflammation-related complications (Fayez et al., 2023). Previously, we identified several vital phytochemicals, including 2-methoxy-4-vinylphenol, gitoxigenin, azafrin, and betulin, through GC–MS analysis of BiE (Anjum et al., 2024), which have been reported to have anti-inflammatory effects (Asami et al., 2023). In the same study, we also reported no observable adverse effects after oral administration of BiE at 2 g/kg in mice (Anjum et al., 2024). In the present study, BiE inhibited albumin denaturation and RBC hemolysis in a dose-dependent manner, with effects that are comparable to those of diclofenac sodium. Inflammation promotes protein denaturation and cell membrane rupture, whereas phytochemicals that prevent both processes indicate anti-inflammatory activity. COX-2 is an inducible form of the cyclooxygenase enzyme, which is expressed in response to inflammatory and related pathological stimuli. COX-2 and 5-LOX enzymes synthesize inflammatory mediators, prostaglandins, and leukotrienes. The inhibition of these enzymes is a key target for controlling inflammation (Chaaban et al., 2024). In addition to inflammation, it has been reported that COX-2 products also contribute to cardiac remodeling after MI (LaPointe et al., 2004). BiE potently blocked the COX-2 enzyme compared to the 5-LOX enzyme. The COX-2 inhibitory activity of saponins isolated from *B. indica* was previously described; however, our study observed greater potency (Othman et al., 2022). The anti-inflammatory potential of BiE was further established through various *in vivo* experiments, including those induced by carrageenan, histamine, and serotonin. Histamine and serotonin administration caused vasodilation and increased vascular permeability, resulting in the leakage of cellular components, which leads to edema and acute inflammation. The administration of carrageenan into an animal’s paw induces the biphasic inflammatory response by increasing the formation of prostaglandins, serotonin, kinins, histamine, leukotrienes, and cytokines such as TNF-α, IL-1β, and IL-6 (Lopes et al., 2020). We observed that pretreatment with BiE inhibited carrageenan-, histamine-, and serotonin-induced inflammation (Figure 1). The percentage of inflammation inhibition increased with time in all the treated groups, up to 6 h, except in the group treated with BiE 30 mg/kg in carrageenan- and serotonin-induced inflammation. BiE at 300 mg/kg showed comparable effects to standard drugs (piroxicam, loratadine, and cyproheptadine), supporting its anti-inflammatory potential. Other plants of the same genus, *Bassia scoparia* and *Bassia eriophora*, have also demonstrated anti-inflammatory effects (Abtulov et al., 2020; Musa et al., 2016). In previous studies, reduced tissue levels of TNF-α and IL-6 were observed in carrageenan-induced inflammation inhibition. These cytokines are also involved in acute MI; such acute anti-inflammatory effects can help to reduce myocyte injury (Xiang et al., 2023).

Recent studies have demonstrated that plants with anti-inflammatory properties can help mitigate post-MI injury; therefore, they are being investigated for their cardioprotective potential. ISO, a β-receptor agonist, increases stress in the myocardium at supramaximal doses, which mimics the effects of acute cardiac infarction in humans. Auto-oxidation of ISO produces free radicals, ROS, lipid peroxidation, and Ca^2+^ overload during cardiac injury, which leads to inflammation, cell necrosis, and apoptosis (Zhang et al., 2023). Heart tissues were stained with TTC, and a large unstained, whitish infarcted area was observed in the ISO group, indicating myocardial injury (Figure 2). Signs of injury such as neutrophil infiltration, vacuolation of cardiomyocytes, broken cardiac fibers, and hyperchromatic nuclei were also observed in H&E-stained cardiac tissue histology (Figure 5). These injuries elevated cardiac biomarkers, including cTnI, CKMB, LDH, and AST, with a significant (*p* < 0.01) increase in serum levels observed in the ISO group compared to that in the normal group (Figure 3). Pretreatment of animals with different doses of BiE (30, 100, and 300 mg/kg) attenuated the cardiac injury caused by ISO and reduced the infarction size and serum levels of these cardiac biomarkers. BiE treatment decreased cTnI levels, which is a highly sensitive and specific cardiac marker, at all doses, indicating its cardioprotective effect. Furthermore, a reduced number of hyperchromatic nuclei, cardiomyocyte vacuolation, neutrophil infiltration, and densely arranged myofibrils in the BiE-treated groups also showed that *B. indica* hindered ISO-induced cardiac injury (Figure 5). These results were similar to previously reported cardioprotective effects of azafrin, gitoxigenin, and betulin phytochemicals isolated from different plants (Hashimoto et al., 1986; Shah and Gandhi, 2024; Yang et al., 2018). These phytochemicals have also been identified in BiE, as reported in our previous study (Anjum et al., 2024).

During the development of MI, an inflammatory response is triggered by pro-inflammatory cytokines, including TNF-α and IL-1β, which are produced in response to NF-κB activation. These interleukins further exacerbate inflammation by producing IL-6 and are involved in the formation of free radicals (ROS), which subsequently intensify cardiac injury (Mehmet et al., 2022). We also observed a significant increase (*p* < 0.001) in the expression of pro-inflammatory cytokines (TNF-α and IL-1β) in the ISO group compared to that in the normal group. In contrast, the level of the anti-inflammatory cytokine IL-10 was decreased (*p* < 0.05). Elevated pro-inflammatory cytokines indicated that ISO-induced cardiac injury also involved inflammation (Figure 4). Similar results were observed in the mRNA expression of pro-inflammatory and anti-inflammatory cytokines. A dose-dependent and significant (*p* < 0.01) decrease in gene and protein expression of TNF-α and IL-1β was observed in BiE-treated groups. However, the effect of BiE treatment on anti-inflammatory mediators was not as prominent as that on pro-inflammatory cytokines, as no significant difference was observed in the IL-10 levels at 30 and 100 mg/kg. These changes in inflammatory cytokines suggest that the cardioprotective effect of BiE is at least partly mediated through the modulation of inflammatory pathways. These results are in line with the findings of previous studies and may be attributed to BiE COX-2 and 5-LOX enzyme inhibition activity (Anajirih et al., 2024; Wu et al., 2024).

An uncontrolled inflammatory response in MI intensifies tissue damage by activating necrotic and apoptotic mechanisms in cardiomyocytes. BCL-2 protein family members, including BAX and BCL-2, modulate the intrinsic apoptotic process (Ali et al., 2023; Shah nd Gandhi, 2024). Increased expression of the BCL-2 protein promotes myocyte survival by enhancing its resistance to apoptosis, a phenomenon that is being widely studied. Cardioprotective agents such as rosuvastatin and quercetin have been reported to decrease apoptosis during cardiac protection by decreasing BAX and increasing BCL-2 protein expression (Sultan et al., 2022). BiE treatment also modulated apoptotic signaling, as reflected by reduced BAX and increased BCL-2 expression, suggesting a shift toward cell survival. This aligns with reports on cardioprotective phytochemicals, such as flavonoids, that reduce apoptosis in myocardial injury models (Figure 4).

The decrease in inflammatory cytokines and apoptosis corresponds to the gene expression of the COX-2 enzyme and NF-κB in cardiac tissue samples. BiE treatment decreased the expression of COX-2 and NF-κB compared to that in the ISO group (Figure 4). It has been observed in several studies that the inhibition of COX-2 and NF-κB decreases the production of pro-inflammatory mediators (IL-1β and TNF-α), which results in the reduction of inflammation and, consequently, cardiac injury (Hassanen et al., 2024). In addition to inflammation, several studies suggested that COX-2 increased the interstitial collagen content and fibroblast proliferation in cardiac cells after MI. The inhibition of COX-2 decreases the collagen content by suppressing inflammation and fibroblast proliferation, ultimately improving cardiac contractility. Previous studies have reported that phytochemicals such as flavonoids and phenolic acids decreased inflammation by attenuating COX-2 and NF-κB, which prevent the adverse cardiac remodeling in MI (LaPointe, et al., 2004). UHPLC-MS/MS analysis of BiE tentatively identified several plant-derived metabolites, including kaempferol 3-glucoside-7-sophoroside, kaempferol 3-rutinoside-7-sophoroside, kaempferol 3-(2G-glucosylrutinoside), ferulic acid, robinetin 3-rutinoside, (3S,7S)-jasmonic acid, and luteolin 7-rhamnosyl-(1->6)-galactoside. These metabolites were previously separated from different plants and shown to have anti-inflammatory activity (Adeyi et al., 2023; Fang et al., 2022; Kim et al., 2013; Kim et al., 2012) and mitigate cardiac injury (Abdulaal et al., 2024; Bekheit et al., 2025; Hua et al., 2022; Lee et al., 2018) through multiple pathways, including the COX-2, NF-κB, Nf2, JAK-STAT, NLRP3 and caspase-1 pathways. For instance, ferulic acid, a polyphenol found in many plants, offers cardiac protection by reducing inflammation, activating Nrf2 signaling, and improving cardiac mechanical function (Pandi et al., 2022). Similarly, luteolin is a commonly found plant flavone, which offers cardio protection by suppressing apoptosis through the upregulation of the AKT pathway, reducing oxidative stress by upregulating HO-1, and downregulating the MAPK pathway (Luo et al., 2017). In the context of these reported studies, the findings of this study suggest that *B. indica* exerts anti-inflammatory effects that attenuate ISO-induced cardiac injury by modulating NF-κB, COX-2, and BAX/BCL-2 pathways.

### 4.1 Limitations of the study

In the present study, we did not assess the functional parameters of the heart (i.e., BP, ECG, and HR) due to the unavailability of an in-house facility. Additionally, we did not assess the downstream levels of caspase-3 in our study, which is considered a limitation of the study.

### 4.2 Prospects of the study

We described that *B. indica* can mitigate acute MI by its anti-inflammatory effects, which corroborate the traditional use of this medicinal plant. However, further detailed studies are required to describe the long-term efficacy, other possible mechanisms, and therapeutic effects of the isolated phytochemicals.

## 5 Conclusion

*B. indica* contains several cardioprotective phytochemicals with reported anti-inflammatory and cardioprotective effects. It suppresses inflammation and apoptosis through the NF-κB and BAX/BCL-2 pathways. This anti-inflammatory and antiapoptotic activity of *B. indica* attenuates ISO-induced cardiomyocyte injury, describing its potential for use in inflammatory and cardiac disorders.

## Data Availability

The original contributions presented in the study are included in the article/Supplementary Material, further inquiries can be directed to the corresponding author.
