# Supplementary material for: Cardioprotective effects of Bassia indica via NF-κB and BCL-2/BAX modulation in isoproterenol-induced myocardial injury
Source: Front Pharmacol. 2025 Oct 14;16:1628715. doi: 10.3389/fphar.2025.1628715 (PMC12558898; doi:10.3389/fphar.2025.1628715)
Supplement: Supplementary file 1 [file DataSheet1.docx]

Supplementary Material

# Operational parameters of MS

The following operational parameters were used for MS: gas temperature 300 °C, drying gas rate of 10 L/min., pressure of nebulizer gas is 45 psi, and capillary voltage of 3500 V for negative ions and 4000 V for positive ions, skimmer voltage of 65 V, fragmentor voltage of 125 V, OCT 1 RF Vpp of 750V.

# Supplementary Figures and Tables

## Supplementary Table

**Supplementary Table** **1.** List of primers used for qPCR.

| **Marker** | **Sequence (5'->3')** | **Reverse /Forward** | **Tm** | **GC%** | **Product length (bp)** | **Gene Accession Number** |
| --- | --- | --- | --- | --- | --- | --- |
| **Cox-2** | ACACACTCTATCACTGGCACC | Forward | 59.72 | 52.38 | **274.00** | **NM_011198.5** |
|  | TTCAGGGAGAAGCGTTTGC | Reverse | 58.37 | 52.63 |  |  |
| **NF-kB** | GCTGCCAAAGAAGGACACGACA | Forward | 63.56 | 54.55 | **131.00** | **NM_001410442.1** |
|  | GGCAGGCTATTGCTCATCACAG | Reverse | 61.64 | 54.55 |  |  |
| **IL-10** | AAGGCAGTGGAGCAGGTGAA | Forward | 61.71 | 55.00 | **159.00** | **NM_010548.2** |
|  | CCAGCAGACTCAATACACAC | Reverse | 61.71 | 55.00 |  |  |
| **Bax** | AGGATGCGTCCACCAAGAAGCT | Forward | 64.24 | 51.65 | **103.00** | **NM_007527.4** |
|  | TCCGTGTCCACGTCAGCAATCA | Reverse | 64.42 | 51.12 |  |  |
| **TNF-α** | ATGGGCTCCCTCTCATCAGT | Forward | 60.03 | 55.00 | **106.00** | **NM_013693.3** |
|  | GCTTGGTGGTTTGCTACGAC | Reverse | 60.03 | 55.23 |  |  |
| **GAPDH** | GACTCCACTCACGGCAAATTC | Forward | 59.50 | 52.38 | **171.00** | **NM_001411843.1** |
|  | TCTCCATGGTGGTGAAGACA | Reverse | 58.30 | 52.12 |  |  |
| **Bcl-2** | CCTGTGGATGACTGAGTACCTG | Forward | 59.83 | 47.55 | **123.00** | **NM_009741** |
|  | AGCCAGGAGAAATCAAACAGAGG | Reverse | 60.56 | 47.83 |  |  |
| **IL-1β** | CACCTCTCAAGCAGAGCACAG | Forward | 60.94 | 54.55 | **79.00** | **NM_031512.2** |
|  | GGGTTCCATGGTGAAGTCAAC | Reverse | 69.11 | 54.28 |  |  |

**Table S2.** Speculative BiE metabolites identified in UHPLC-MS/MS in negative and positive mode.

| Sr. No | RT (min) | Metabolites | MF | MW  (g/mol) | Base peak  (m/z) |
| --- | --- | --- | --- | --- | --- |
| 1 | 0.95 | 3-Furoic acid | C_5_H_4_O_3_ | 112.0 | 111.0 |
| 2 | 6.30 | Benzenebutanoic acid, 2-hydroxy-3,4-dimethoxy-6-methyl-5-(sulfooxy)- | C_13_H_18_O_9_S | 350.0 | 349.0 |
| 3 | 7.80 | 4-Hydroxyphenylpyruvic acid | C_9_H_8_O_4_ | 180.1 | 179.0 |
| 4 | 7.95 | 4-(3-Methylbut-2-enyl)-L-tryptophan | C_16_H_20_N_2_O_2_ | 272.3 | 307.1 |
| 5 | 7.96 | N-Feruloylglycine | C_12_H_13_NO_5_ | 251.2 | 250.0 |
| 6 | 8.33 | Dihydroferulic acid 4-O-glucuronide | C_16_H_20_O_10_ | 372.3 | 371.1 |
| 7 | 8.79 | Robinetin 3-rutinoside | C_27_H_30_O_16_ | 610.5 | 609.1 |
| 8 | 9.11 | N2-Malonyl-D-tryptophan | C_14_H_14_N_2_O_5_ | 290.2 | 291.0 |
| 9 | 9.66 | Ligulatin B | C_17_H_22_O_5_ | 306.4 | 341.1 |
| 10 | 9.665 | Scandenin | C_26_H_26_O_6_ | 434.5 | 469.1 |
| 11 | 9.93 | p-Salicylic acid | C_7_H_6_O_3_ | 138.1 | 137.0 |
| 12 | 10.67 | Carpelastofuran | C_30_H_34_O_8_ | 522.6 | 521.2 |
| 13 | 11.97 | Durupcoside B | C_47_H_74_O_18_ | 927.1 | 925.4 |
| 14 | 15.86 | 12-oxo-10Z-octadecenoic acid | C_18_H_32_O_3_ | 296.4 | 295.2 |

## Supplementary Figures

**
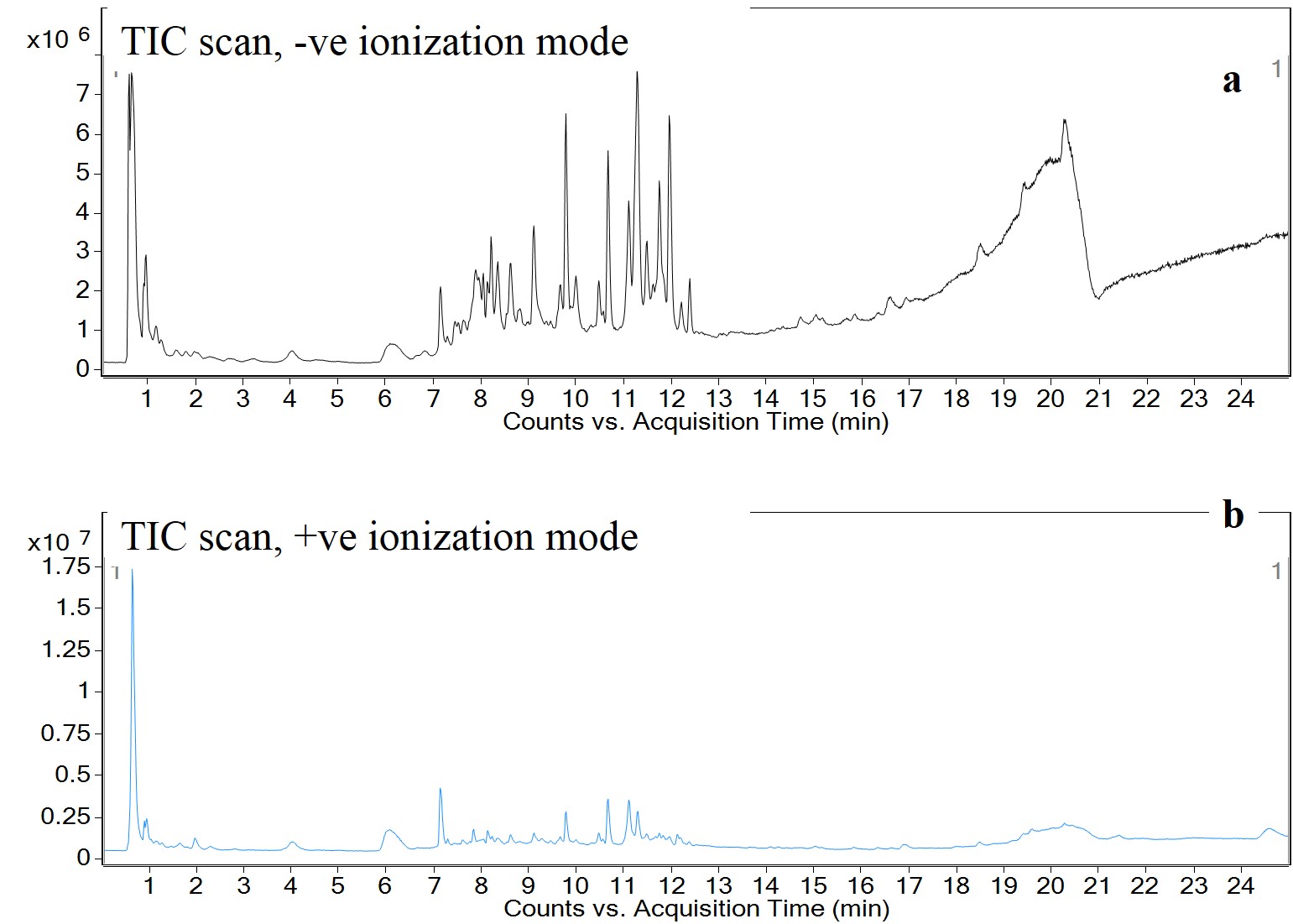
**

**Supplementary Figure 1.** Total ion chromatographs (TIC) of UHPLC-MS/MS of Bi.Cr **(a)** negative ionization mode **(b)** positive ionization model


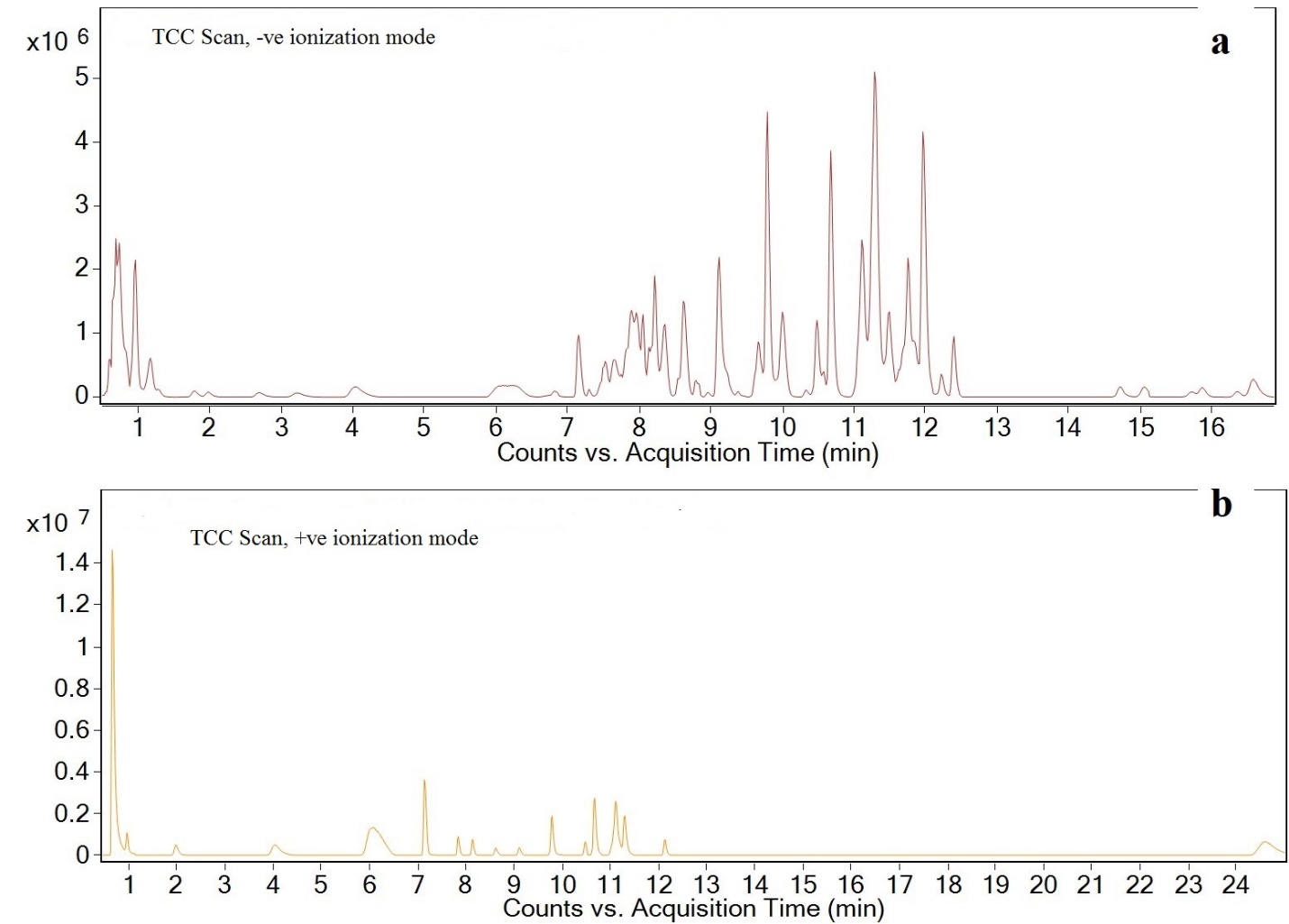


**Supplementary Figure 2.** Total compound chromatographs (TIC) of UHPLC-MS/MS of Bi.Cr **(a)** negative ionization mode **(b)** positive ionization model
